# Photocatalytic Properties of Graphene/Gold and Graphene Oxide/Gold Nanocomposites Synthesized by Pulsed Laser Induced Photolysis

**DOI:** 10.3390/nano10101985

**Published:** 2020-10-07

**Authors:** Li-Hsiou Chen, Huan-Ting Shen, Wen-Hsin Chang, Ibrahim Khalil, Su-Yu Liao, Wageeh A. Yehye, Shih-Chuan Liu, Chih-Chien Chu, Vincent K. S. Hsiao

**Affiliations:** 1 Department of Pulmonary Medicine, Taichung Tzu Chi Hospital, Buddhist Tzu Chi Medical Foundation, Taichung 427, Taiwan; chenindy@tzuchi.com.tw (L.-H.C.); ryenhat@tzuchi.com.tw (H.-T.S.); 2 Department of Medical Applied Chemistry, Chung Shan Medical University, Taichung 40201, Taiwan; dadada5202008@gmail.com; 3 Department of Applied Materials and Optoelectronic Engineering, National Chi Nan University, Nantou 54561, Taiwan; ikhalilcu@gmail.com; 4 Nanotechnology & Catalysis Research Centre (NANOCAT), Institute for Advanced Studies, University of Malaya, Kuala Lumpur 50603, Malaysia; 5 Department of Electrical Engineering, National Chi Nan University, Nantou 54561, Taiwan; suyu@ncnu.edu.tw; 6 Department of Health Diet and Industry Management, Chung Shan Medical University, Taichung 40201, Taiwan; liou@csmu.edu.tw; 7 Department of Medical Education, Chung Shan Medical University Hospital, Taichung 40201, Taiwan

**Keywords:** graphene, graphene oxide, gold, nanocomposite, photolysis, photocatalysis

## Abstract

Graphene (Gr)/gold (Au) and graphene-oxide (GO)/Au nanocomposites (NCPs) were synthesized by performing pulsed-laser-induced photolysis (PLIP) on hydrogen peroxide and chloroauric acid (HAuCl_4_) that coexisted with Gr or GO in an aqueous solution. A 3-month-long aqueous solution stability was observed in the NCPs synthesized without using surfactants and additional processing. The synthesized NCPs were characterized using absorption spectroscopy, transmission electron microscopy, Raman spectroscopy, energy dispersive spectroscopy, and X-ray diffraction to prove the existence of hybrid Gr/Au or GO/Au NCPs. The synthesized NCPs were further evaluated using the photocatalytic reaction of methylene blue (MB), a synthetic dye, under UV radiation, visible light (central wavelength of 470 nm), and full spectrum of solar light. Both Gr/Au and GO/Au NCPs exhibited photocatalytic degradation of MB under solar light illumination with removal efficiencies of 92.1% and 94.5%, respectively.

## 1. Introduction

With the rapid progress in industrialization, the wastewaters containing synthetic dyes, such as methylene blue (MB), are not easily degradable and potentially toxic. Hence, these dyes cause adverse effects to the aquatic organisms and humans, even when present in minute concentrations [1,2]. Hence, an effective treatment that enables the removal or degradation of synthetic dyes present in industrial effluents before discharging the effluents into the environment should be developed urgently for minimizing environmental pollution. To date, various techniques, such as adsorption, coagulation, aerobic oxidation, membrane filtration, chemical precipitation, ozonization, and photocatalysis have been employed for the removal of dyes from industrial wastewater [3,4,5,6,7]. Of these techniques, photocatalysis has emerged as a promising green technique and been widely used for the degradation of dyes because of its easy operation process, low cost, and energy conservation ability. Moreover, under the irradiation of light, particularly solar light, the photocatalytic treatment yields CO_2_, H_2_O, and other nontoxic compounds. A complete degradation of the dyes occurs at a large extent under ambient conditions of temperature and pressure [8,9,10]. However, the photocatalyst itself remains unchanged during the treatment process. Moreover, the process does not require any consumable chemicals, and landfill is not required for sludge disposal because no residue of the original material remains [9,11].

Of the different types of heterogeneous photocatalysts, TiO_2_ has been the most studied [12,13]. Other semiconductor materials, such as ZnO, CuO, CeO_2_, SnO_2_, CdS, and ZnS, have also been used for the photocatalytic degradation and removal of organic dyes [14,15]. Semiconductor materials exhibit an excellent photoinduced redox reaction because of their specific electronic structure (filled valence and empty conduction bands). Hence, semiconductor materials are materials of choice for the photocatalytic removal of organic and synthetic dyes [16]. However, suitable matrix materials, such as zeolite, silica, activated carbon, carbon nanotube, graphene (Gr), and graphene oxide (GO), have been reported to enhance the photocatalytic degradation efficiency [13]. Gr has a two-dimensional planar structure comprising single-layer sp^2^-bonded carbon atoms arranged in a honeycomb lattice structure, zero band-gap semiconductor properties with a large surface area, high charge carrier mobility, high adsorption capacity, and excellent electron transfer rate [3,17,18,19].

Gr and GO have extraordinary properties and have been widely used in various applications since their discovery in 2004. Moreover, Gr-based nanocomposites (NCPs), such as Gr-metal and Gr-metal-oxide NCPs, have been tested for exhibiting more advantageous properties than those of the individual material alone to explore a higher number of applications and to enhance the expected outcome or efficiency. Gr/gold (Au) and GO/Au NCPs are the most commonly used hybrid NCPs among the different hybrid NCPs because of the remarkable features of Au nanoparticles (NPs), such as higher chemical stability, catalytic activity, easy surface functionalization, and biocompatibility [20]. Gr reveals the improved activities as photocatalysis to remove the dyes. For example, Yang et al. synthesized a porous TiO_2_/Gr composite that presented a higher degradation rate (6.5 times) of MB in commercial P25 [2]. Similarly, the TiO_2_/reduced-GO (rGO) NCPs were prepared using different amounts of Gr (1–20%) through two different synthesis routes. When 10% Gr was added in TiO_2_/rGO NCPs during the fabrication process, the highest photocatalytic activity was observed toward MB. This activity attained a value of 93% for the NCPs prepared using the sol-gel method that is followed by the hydrothermal treatment and a value of 82% for the NCPs prepared using the hydrothermal route only [13]. Some other Gr-based binary NCPs, such as the GO/TiO_2_ hydrogel [12], TiO_2_-doped calcined mussel shell [11], Gr/SnO_2_ [15], Gr/ZnO [21], ZnO-decorated GO [22], Gr/CeO_2_ [14], and Bi_2_MoO_6_/rGO aerogel NCPs [9] were evaluated for the photocatalytic degradation of MB under visible light illumination. Moreover, ternary composites, such as rGO/Fe_3_O_4_/TiO_2_ [3], Fe_2_O_3_/Gr/CuO [5], GO/mesoporous TiO_2_/Au [10], and MoO_3_/Fe_2_O_3_/rGO NCPs [1] were investigated. However, few discussions on photocatalytic properties of Gr/Au or GO/Au NCPs were proposed [23].

**Figure 1:**
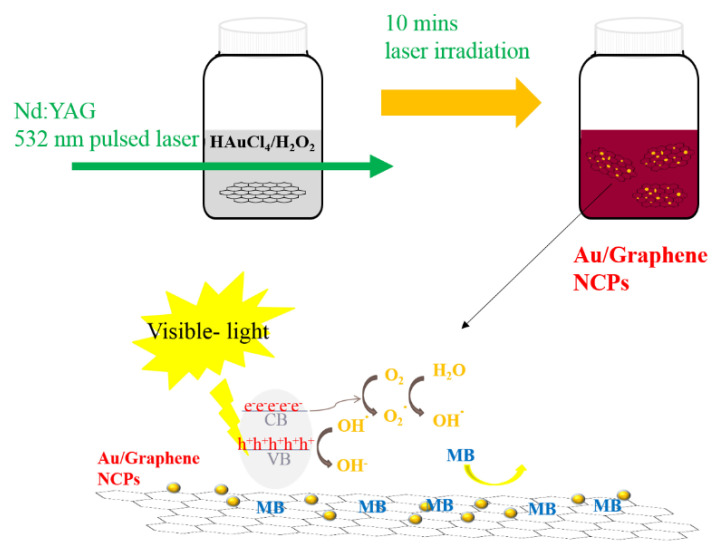
Schematic of the pulsed-laser-induced photolysis (PLIP) method to fabricate Au/Graphene nanocomposites (NCPs) used in visible photocatalysis.

Thus far, many fabrication methods, such as some green synthesis approaches, have been reported for the synthesis of Gr/Au or GO/Au NCPs [24,25]. The most common approach is the chemical reduction method that use different chemical reductants [26,27]. A major disadvantage of using a chemical reducing agent is the presence of this reducing agent in the final composite even after washing multiple times, thus considerably limiting their application. By contrast, Gr/metal or GO/metal NCP synthesis performed using photochemical or photolysis reaction has many advantages. For instance, avoiding the use of toxic chemical reducing agents, which are generated from reducing or capping agents, could provide intended applications, could prevent negative influences, and can present better control of Au over Gr or GO sheets without requiring a high temperature [28]. Pulsed laser-induced photolysis (PLIP) has been used for fabricating pure Au NPs [29], Au NP micelles [30,31], and the Au/Ag NP alloy [32]. Instead of using an Au target with pulsed laser ablation to fabricate Au NP, NCPs or the Au NP hybrid NCPs [33,34,35,36], chloroauric acid (HAuCl_4_) can be used as a precursor and photodecomposing agent to obtain Au^3+^. This ion is later reduced by the photon, and Au NPs are generated under the high-energy pulsed laser [29]. Because of the unique properties of ultra-short pulse duration and ultra-high peak power intensity, the pulsed-laser-induced synthesis technique, such as pulsed laser ablation and PLIP, is considered a clean and prompt technique to reduce metal ions into NPs without using any other chemical reagent. However, the long-term stability of synthesized Au NPs hinders their practical application. In this study, we reported the clean synthesis of Gr/Au and GO/Au NCPs by using the photolysis technique induced using a nanosecond pulsed laser operated at a 532 nm wavelength, as shown in Figure 1. A one-pot synthesis was conducted in which all precursors, HAuCl_4_, hydrogen peroxide (H_2_O_2_) and Gr or GO, were dissolved or dispersed in an aqueous solution. Both H_2_O_2_ and HAuCl_4_ undergo the photolysis process and generate Au^3+^ and HO–O^●^. The photolysis-induced Au^3+^ was further reduced by HO–O^●^, which is an effective one-electron reducing agent [37]. Gr/Au and GO/Au NCPs with long-term stability in aqueous solution could be synthesized by adding Gr or GO into the precursor solution. Different H_2_O_2_ and HAuCl_4_ amounts were used to optimize the stability and photocatalytic properties of Gr/Au and GO/Au NCPs under different light exposure conditions, dark, UV, visible, and solar light.

## 2. Materials and Methods

### 2.1. Materials

Gr and GO powders were purchased from Ritedia Corporation (Hsinchu, Taiwan) and Tokyo Chemical Industry (Tokyo, Japan), respectively. MB (C_16_H_13_N_3_SCl) was obtained from Katayama Chemical Company (Osaka, Japan). Tetrachloroauric (III) acid trihydrate (HAuCl_4_·3H_2_O) and H_2_O_2_ (35 wt.% solution in water) were obtained from Acros Organics (Geel, Belgium). Milli-Q water (18.2 MΩ cm) was used as the aqueous solution throughout the study and was prepared in house.

### 2.2. Characterization

X-ray diffraction (XRD) patterns of all the synthesized samples were obtained in the 2*θ* range of 0–90° on a high-resolution X-ray diffractometer (Bruker AXS Gmbh, Karlsruhe, Germany) The elemental analysis of the samples was performed through X-ray energy dispersive spectrometry (EDS) (Bruker AXS Gmbh, Karlsruhe, Germany). The morphology, particle size, and distribution of the Au NPs on Gr or GO sheets were investigated through transmission electron microscopy (TEM) on a JEM-2100 transmission electron microscope (JEOL, Tokyo, Japan). UV/Vis spectrophotometry was performed using a UV/Vis spectrophotometer (GENESYS 10S; Thermo Scientific, Waltham, MH, USA) to justify GO/Au NP and Gr/Au NP NCP syntheses and evaluate degradation percentage of all samples under different conditions. Raman spectra were obtained using a microRaman system (LabRAM HR800; HORIBA Jobin Yvon, Northampton, UK) with a helium–neon laser as the excitation source operating at a 633-nm wavelength and a 40× objective lens. The other instruments are as follows: delta ultrasonic cleaner (DC200H; Delta, Taipei, Taiwan), bench-top centrifuge (Velocity 14; Dynamica, Hong Kong), 2996 photodiode-array detector (PDA; Waters, MA, USA), solar simulator (Prosper OptoElectronics, Hualien, Taiwan), and universal centrifuges (Hermle LaborTechnik, Wehingen, Germany).

### 2.3. Gr/Au and GO/Au NCP Syntheses

Gr/Au and GO/Au NCPs were synthesized using pulsed-laser-induced photolysis. For synthesis, different concentrations and amounts of HAuCl_4_·3H_2_O were added to a 7 mL transparent glass bottle. Then, a different amount of H_2_O_2_ at fixed concentration of 10 mM and fixed 1 mL of Gr or GO suspension (0.1 mg/mL) was added to the bottle. The details of each sample fabricated using different experimental conditions are shown in Table S1. The aqueous suspension was then irradiated for 10 min with a pulsed Q-switch Nd:YAG laser (LS-2137U; LOTIS TII, Minsk, Belarus) with a wavelength of 532 nm, pulse duration of 6–7 ns, pulse repetition rate of 10 Hz, and fluence of approximately 37 mJ/cm^2^. The laser beam was delivered in the middle of the precursor solution to ensure a homogenous light exposure to the sample. The precursor solution changed from light grey to reddish purple after 10 min. The pulsed-laser-treated solutions containing NCPs which have long-time stability were used for photocatalytic measurement without additional treatment.

### 2.4. Photocatalytic Activity Test

The photocatalytic activity of the synthesized binary NCPs was evaluated based on the degradation of MB in a homemade dark room setup, under UV light emitting diode (LED) (365 nm wavelength, 100 mW/cm^2^), under laser diode (LD) (470 nm wavelength, 500 mW/cm^2^), and under solar simulator (Xenon lamp, 50 W/cm^2^). For the evaluation of photocatalytic degradation, 50 mL of 10 mg/L MB solution was transferred into a sample bottle. Then, 10 mg of the respective synthesized sample composite were added into the bottle, and the sample composite was uniformly dispersed by magnetic stirring at 550 rpm for 5–10 min. Each sample suspension was then tested for photocatalytic activity by placing in a dark room and under UV LED, visible LD, and solar light sources. The absorption spectra were recorded at different time intervals. The photocatalytic degradation rate and removal efficiency of MB were calculated by measuring the absorbance maximum value of the treated solutions at 664 nm by using the following equation: (*A*_0_ − *A_t_*)/*A*_0_ × 100%, where *A*_0_ and *A_t_* are the initial and final absorbance values of MB solution at the specified time, respectively.

## 3. Results and Discussion

### 3.1. Laser-Induced Photolysis and Formation of Au NPs

Gr/Au and GO/Au NCPs were synthesized by performing Nd:YAG laser-induced photolysis of HAuCl_4_ in the presence of aqueous H_2_O_2_ as a reducing agent and with the addition of Gr or GO. In the reaction mechanism, on laser excitation, HAuCl_4_ dissociates into AuCl_3_^−^ and H_2_O_2_ undergoes the photolytic process and generates an effective one-electron reducing agent HO–O^●^ [37]. AuCl_3_^−^ is further reduced to Au° in the presence of metal ions through the following three steps. Finally, Au atoms aggregated to form Au NPs supported on the Gr or GO nanosheets. Hence, the reaction protocol is a fast and clean approach to generate NCPs by using H_2_O_2_ as a reducing agent in an aqueous solution.

**Step 1: Au^3+^ Formation**

(1) $${\mathrm{HAuCl}}_{4}\to H^{+}+\mathrm{AuCl}4_{4}^{-}$$

(2) $${\mathrm{AuCl}}_{4}^{-}\overset{hv}{\to }{\mathrm{Au}}^{3+}{\mathrm{Cl}}_{3}^{-}+{\mathrm{Cl}}^{•}$$

**Step 2: H_2_O_2_ Photolysis**

(3) $$H_{2}O_{2}\overset{hv}{\to }2{\mathrm{HO}}^{•}$$

(4) $${\mathrm{HO}}^{•}+H_{2}O_{2}\to \mathrm{HO}-O^{•}+H_{2}O$$

**Step 3: Au NP Generation**

(5) $$\mathrm{HO}-O^{•}+{\mathrm{Au}}^{3+}\to {\mathrm{Au}}^{2+}+O_{2}+H^{+}$$

(6) $$\mathrm{HO}-O^{•}+{\mathrm{Au}}^{2+}\to {\mathrm{Au}}^{+}+O_{2}+H^{+}$$

(7) $$\mathrm{HO}-O^{•}+{\mathrm{Au}}^{+}\to {\mathrm{Au}}^{0}+O_{2}+H^{+}$$

(8) $${\mathrm{nAu}}^{0}\to \mathrm{AuNPs}$$

### 3.2. Stability of Synthesized NCP

UV/Vis spectroscopy was conducted to justify the formation of Au NPs either in the NP or NCP form. Upon laser excitation, the HAuCl_4_ solution co-existing with H_2_O_2_ was converted to Au NPs. This conversion was indicated by the color change to a reddish-purple color and the shifting of the UV/Vis absorption peak from 290 nm (HAuCl_4_ solution) to 526 nm (Figure S1). Here we used different combination of the concentration and the amount of HAuCl_4_ and H_2_O_2_ to fabricate Gr/Au and GO/Au NCPs. Some experimental conditions failed to fabricate NCPs. Table S1 shows the experimental results indicating higher concentration of HAuCl_4_ is not suitable to fabricate NCPs, for example, the sample number AuG6, AuG7, AuGO6 and AuGO7 of 4 mM concentration. The interesting result from sample number AuG3 and AuGO3 shows the total volume of precursors has to be controlled in a small volume (<3.5 mL) to achieve the success fabrication of NCPs. This result may be attributed to the small exposure area from the pulsed laser.

**Figure 2:**
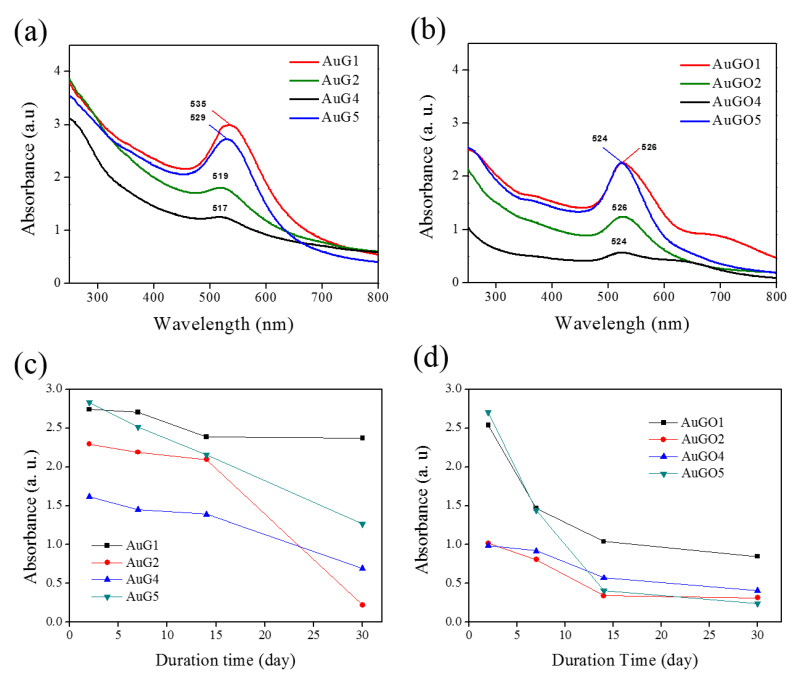
Characteristic absorption spectra of (**a**) Gr/Au NCPs and (**b**) GO/Au NCPs. Stability evaluation of (**c**) Gr/Au NCPs, and (**d**) GO/Au NCPs by recording the peak absorption of samples at different time intervals at room temperature.

Similarly, the Gr/Au and GO/Au NCPs were characterized using the UV/Vis absorption spectra to identify the existence of Au NPs with the characteristic absorption peak at 526 and 535 nm, respectively (Figure 2a,b). All samples show red absorption peaks, and the GO/Au NCPs (AuGO1 and AuGO4) show extra absorption at around 700 nm wavelength. The absorbance from the sample solutions was measured continuously to determine the stability of the synthesized Au NPs, Gr/Au NCPs, and GO/Au NCPs, as shown in Figures S2 and S3, separately. The Gr/Au and GO/Au NCPs reveal high stability after 48 h compared with the unstable Au NPs in aqueous solution. Gr/Au NCPs (AuG1) reveal optimal stability with an absorbance value of 2.88 at a wavelength of 537 nm after 48 h, as shown in Figure 2c. This value decreased to 2.46 after 30 days with 2-nm blue shifting (Figure S2). The synthesized Gr/Au NCPs maintain high stability even after 30 days (AuG1 and AuG5); thus, Gr/Au NCPs are considered a better candidate for photocatalysis because of the high stability for a long duration. GO/Au NCPs reveal high stability in 1 week (Figure S3) by observing the characteristic absorbance located in the 537 nm, the same resonance peak as Gr/Au NCP, with only decreasing the absorbance intensity. However, the peak absorbance observed from GO/Au NCPs became even, as shown in Figure 2d, thus indicating a decrease in the amount of Au remaining inside the GO nanosheets.

### 3.3. Characterization of Synthesized NCPs

**Figure 3:**
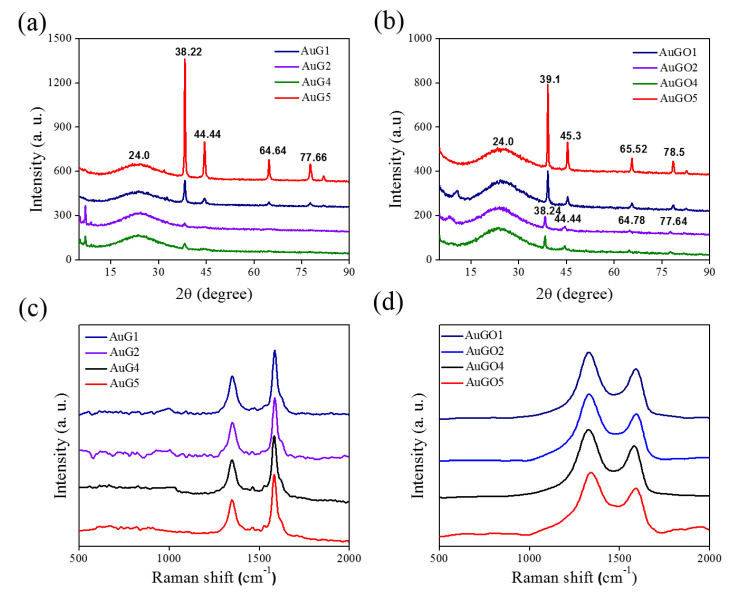
XRD spectra of (**a**) Gr/Au and (**b**) GO/Au NCPs, and Raman spectra of (**c**) Gr/Au and (**d**) GO/Au NCPs.

The XRD spectra of the synthesized Gr/Au NCPs that is presented in Figure 3a revealed four major diffraction peaks at 2*θ* of 38.22°, 44.44°, 64.64°, and 77.66° that correspond to (111), (200), (220), and (311) crystal planes of Au NPs [38], respectively. Moreover, Gr/Au NCPs exhibited another diffraction peak at the 2*θ* of 24° that corresponds to the (002) plane of Gr nanosheets, thus indicating the presence of Gr in the synthesized NCPs [5]. Similarly, XRD spectra of GO/Au NCPs, as shown in Figure 3b, presented the same diffraction peaks with minor red-shifting to 2*θ* of 39.1°, 45.3°, 65.52°, and 78.5°. GO/Au NCPs exhibited a broadened diffraction peak at a 2*θ* of 24° that corresponded to the (002) plane assigned to the Gr material, thus indicating that the starting material GO was reduced to Gr by using a Nd:YAG-induced pulsed laser irradiation at a wavelength of 532 nm [39]. Moreover, the intense peak at 38.22° indicates the preferential growth of Au NPs in the (111) plane. The observed XRD spectra of the synthesized NCPs reveal the successful fabrication of Au NPs over the Gr and GO nanosheets.

The Raman spectra of the Gr/Au NCPs were characterized using a sharp D band at approximately 1350 cm^−1^ and G band at approximately 1585 cm^−1^ (Figure 3c). The D band is attributable to the defect induced features resulting from vibrations of carbon atoms with dangling bonds and corresponds to the breathing mode of k-point phonons of the A_1g_ symmetry. However, the G band refers to the in-plane phonon vibration of sp2 carbon atoms with the E_2g_ symmetry. By contrast, GO/Au NCPs represent the typical Raman spectra with a strong and broad D band [39] at approximately 1330 cm^−1^ and G band at approximately 1590 cm^−1^ (Figure 3d). The observation of Raman spectra also proves the existence of the precursors (graphene, graphene oxide, and chloroauric acid) originally added into the aqueous solution.

**Figure 4:**
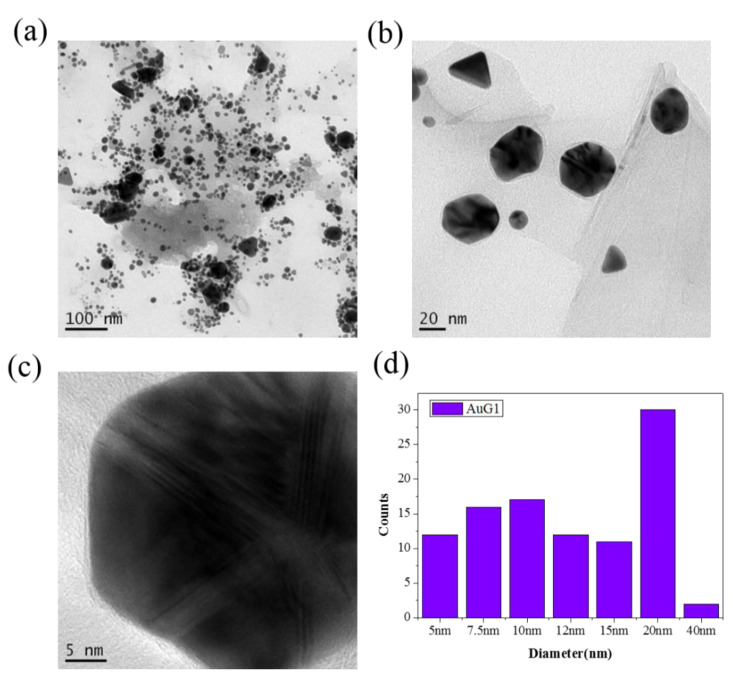
TEM image of different magnification (**a**–**c**) and (**d**) corresponding size distribution of Gr/Au NCPs (Sample AuG1).

The size, morphology, distribution, and elemental compositions of the Gr/Au and GO/Au NCPs were evaluated through TEM and EDS, as shown in Figures S4 and S5. Those findings clearly demonstrate that the NCPs are a combination of Au NPs and Gr or GO materials. Figure 4 shows the typical TEM images and corresponding size distribution of NCPs observed from AuG1 sample. Elemental analysis reveals that the samples comprise gold, carbon, and oxygen. We also evaluate the Au loading by theoretically calculating the amount of added HAuCl_4_ and experimentally observed results from EDS, as shown in Table S2. The evaluations are matched in the fabricated samples using Gr as Au loading materials; however, the use of GO shows interesting results that both the amounts of HAuCl_4_ and H_2_O_2_ have to be the same to get the maximum loading of Au NPs. All TEM images reveal that the Au NPs were distributed uniformly. Moreover, in few cases under agglomerated conditions, the Au NPs were distributed on smooth, almost transparent single- or few-layered wrinkled Gr or GO nanosheets. Hence, TEM images provide a direct evidence of the decoration of Au NPs on planar Gr or GO sheets, which provide high active surface areas for the intended photocatalytic degradation of the dye.

### 3.4. Photocatalytic Activity of Synthesized Materials

**Figure 5:**
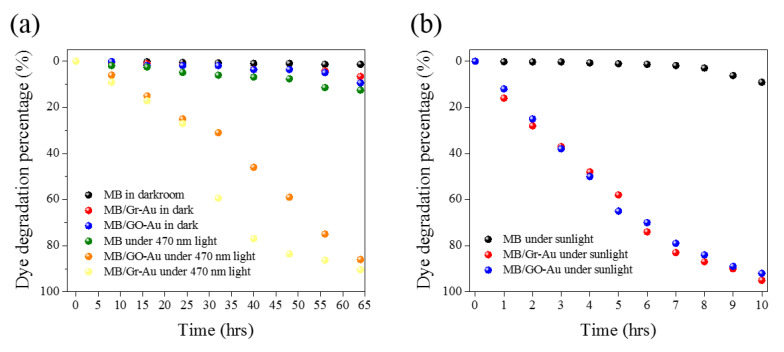
Photocatalytic activity of Gr/Au and GO/Au NCPs in MB solution as a function of the exposure time under (**a**) visible and (**b**) solar light.

The photocatalytic performance of the synthesized and selected Gr/Au and GO/Au NCPs were evaluated through the degradation ability of MB in the aqueous solution under four different conditions—in a darkroom, under UV radiation (wavelength, 365 nm), visible light (wavelength, 470 nm), and simulated solar light (wavelength, 350–915 nm). Aqueous MB solution (10 mg/L) was used as the control. The MB degradation activity of the samples treated in the dark and under UV and visible light was evaluated at an interval of 8 h for a total of 64 h. The samples irradiated with solar light were checked at an interval of 2 h for a total 10 h period. Both composites revealed slight degradation of the dye in the dark-room condition even after storage for a long period of 64 h. The degradation percentages for Gr/Au and GO/Au NCPs were 6.55% and 7.98%, respectively (Figures S6a and S7a, respectively). Hence, the dark-room condition confirmed that the absorption of dye from Gr or GO has no obvious catalytic role in dyes because the degradation ratio is significantly small. The absorption spectra of MB in a dark room, as shown in Figure 5 (black dot), at a gradual time interval did not reveal any change, thus indicating that MB cannot easily degrade by itself. However, the samples treated under UV radiation revealed a gradual increase in the degradation activity with increase in the treatment time and attained a value of 28.18% by using Gr/Au NCPs (Figure S6b) and 21.63% by using GO/Au NCPs (Figure S7b) after 64 h. By contrast, the degradation rate under visible illumination was really convincing and much greater than that under UV radiation. The NCPs under visible light (470 nm) illumination reveal a gradual increase in the degradation activity with increase in the exposure period. The lowest absorption peak was observed at 664 nm after a 64-h treatment period. Hence, the highest degradation percentage was 90.51% for Gr/Au NCPs (Figure S6c) and 86.09% by the GO/Au NCPs (Figure S7c).

The most potential photocatalytic degradation mechanism was achieved by treating the MB solution with NCPs under solar light. The degradation efficiency of the samples was checked at a 1 h interval when placed under a 100 W solar simulator. Both NCPs revealed strong MB degradation activities under solar light with gradual dye removal, thus presenting a declining trend of absorbance maxima at 664 nm with the progress of the exposure period. At the end of the 10 h exposure period, 94.5% of the dye was removed by the activity of Gr/Au NCPs (Figure S6d). However, the MB removal percentage was 92.1% when GO/Au NCPs were used (Figure S7d). With increase in the exposure period, the UV spectra also slightly shifted to the left from 664 nm. Throughout the study, the photocatalytic degradation of the MB, Gr/Au NP showed better degradation ability than GO/Au NP (Figure 5). Comparing the previous studies [23,24] of using similar NCPs to measure their photocatalytic ability, our NCPs required more time to achieve 100% degradation; however, other studies have no discussion regarding the long-term stability of NCPs. Therefore, our synthesized NCPs still show potential use in a hash environment where long-term stability is necessary to achieve photocatalytic ability.

Au NPs were formed and anchored immediately onto the Gr/GO nanosheets by using pulsed-laser-induced photolysis. Hence, the stable, binary NCPs with uniform dispersion of Au NPs were suspended inside the sample solution and actively participated in the MB degradation activity. A Schottky barrier was formed at the interface of Gr and Au particles because of the higher work function of Au than that of Gr. Thus, the degradation rate considerably increased under visible light irradiation [40,41,42,43]. The electrons injected on the Gr surface subsequently moved to Au NPs, which spatially separated MB^+^ and electrons. Thus, the recombination process was delayed. The decrease in electron accumulation on the Gr surface evidently enhanced the continuous electron transfer from MB^●^ to Gr. Moreover, surface adsorbed O_2_ could easily trap the electrons from Au particles to form various reactive oxidative species, such as HO^●^, because Au is an efficient electron donor, thus greatly improving MB degradation. Because of differences between the work functions of Gr (−4.42 eV) and Au NPs (−4.75 eV), direct electron transfer from MB^●^ to the semiconducting Gr or GO enables MB photodegradation [23]. Hence, the possible degradation mechanism of MB by the synthesized NCPs under solar radiation involves the excitation of the dye MB^●^, followed by electron transfer from MB^●^ to Gr or GO. The electrons subsequently move to Au NPs and are trapped using O_2_ to produce HO^●^. The MB^●+^ finally degrades by itself and/or is degraded by HO^●^. Thus, Gr/Au or GO/Au NCPs synthesized through a simple, straightforward PLIP method without requiring a potential reducing agent can be promising agents in the photocatalysis field.

## 4. Conclusions

Gr/Au and GO/Au NCP hybrid photocatalysts were successfully synthesized through a simple, fast, and clean technique by using PLIP. H_2_O_2_, a volatile reductant, was used in this technique during pulsed laser irradiation in the presence of HAuCl_4_ that coexisted with aqueous Gr or GO suspension to generate NCPs within a few minutes with H_2_O and O_2_ generation as the final byproducts. Both Gr/Au and GO/Au NCPs were characterized by the uniform dispersion of Au NPs over GO and Gr sheets. Hence, a planar and large surface area is provided for the photocatalysis of MB. The photocatalytic performances of the synthesized NCPs were evaluated under UV, visible, and solar light. Both Gr/Au and GO/Au NCPs exhibited optimal photocatalytic performance under solar light and exhibited degradation efficiencies of 94.5% and 92.1% after a 10-h exposure period. Therefore, the current study outcome suggests that the NCPs obtained using the green synthesis approach are excellent and stable photocatalysts for the photocatalytic degradation of MB, a common water polluting agent. Hence, these NCPs have a large potential in the practical treatment of dye-contaminated wastewater through an ecofriendly fabrication process.

## Supplementary Materials

The following are available online at https://www.mdpi.com/2079-4991/10/10/1985/s1, Figure S1: Characteristic absorption spectra of HAuCl_4_ when used as an aqueous solution and the corresponding Au NPs, Gr/Au NCPs, and GO/Au NCPs. Figure S2: Characteristic absorption spectra of the corresponding Gr/Au NCPs by observing the absorption spectra of samples at different time intervals at room temperature. The inset shows the photo of the solution after 30 day storage at dark room. Figure S3: Characteristic absorption spectra of the corresponding GO/Au NCPs by observing the absorption spectra of samples at different time intervals at room temperature. The inset shows the photo of the solution after 30 day storage at dark room. Figure S4: Elementary analysis of Gr/Au and GO/Au NCPs. Figure S5: TEM image and corresponding size distribution of Gr/Au and GO/Au NCPs. Figure S6: Change in absorbance of MB when photocatalytic degradation is performed (a) in a darkroom and under (b) UV, (c) visible, and (d) solar light in the presence of Gr/Au NCPs. Figure S7: Change in the absorbance of MB when photocatalytic degradation is performed (a) in a darkroom and under (b) UV, (c) visible, and (d) solar lights in the presence of GO/Au NCPs. Table S1: Experimental conditions of nanocomposites fabricated by PLIP method. Table S2: Theoretical and experimental evaluation of Au loading.
