# Supplementary material for: Photocatalytic Properties of Graphene/Gold and Graphene Oxide/Gold Nanocomposites Synthesized by Pulsed Laser Induced Photolysis
Source: Nanomaterials (Basel). 2020 Oct 7;10(10):1985. doi: 10.3390/nano10101985 (PMC7599496; doi:10.3390/nano10101985)
Supplement: Supplementary file 1 [file nanomaterials-10-01985-s001.pdf]

Supplementary Materials: Photocatalytic Properties of Graphene/Gold and Graphene Oxide/Gold Nanocomposites Synthesized by Pulsed Laser Induced Photolysis

Li-Hsiou Chen [1], Huan-Ting Shen [1], Wen-Hsin Chang [2], Ibrahim Khalil [3,4], Su-Yu Liao [5], Wageeh A. Yehye [4], Shih-Chuan Liu [6], Chih-Chien Chu [2,7,*] and Vincent K. S. Hsiao [3,*]

Table S1. Experimental conditions of nanocomposites fabricated by PLIP method.

Sample Number	Concentration of $HAuCl_4$ (mM)	Amount of $HAuCl_4$ (mL)	Amount of H_2O_2 (mL)	Formation of Nanocomposite
AuG1	0.33	2.5	1	Yes
AuG2	0.33	0.5	0.5	Yes
AuG3	0.33	10	10	No
AuG4	0.33	0.5	1	Yes
AuG5	0.33	2.5	0.5	Yes
AuG6	4	0.5	0.5	No
AuG7	4	0.5	1	No
AuGO1	0.33	2.5	1	Yes
AuGO2	0.33	0.5	0.5	Yes
AuGO3	0.33	10	10	No
AuGO4	0.33	0.5	1	Yes
AuGO5	0.33	2.5	0.5	Yes
AuGO6	4	0.5	0.5	No
AuGO7	4	0.5	1	No

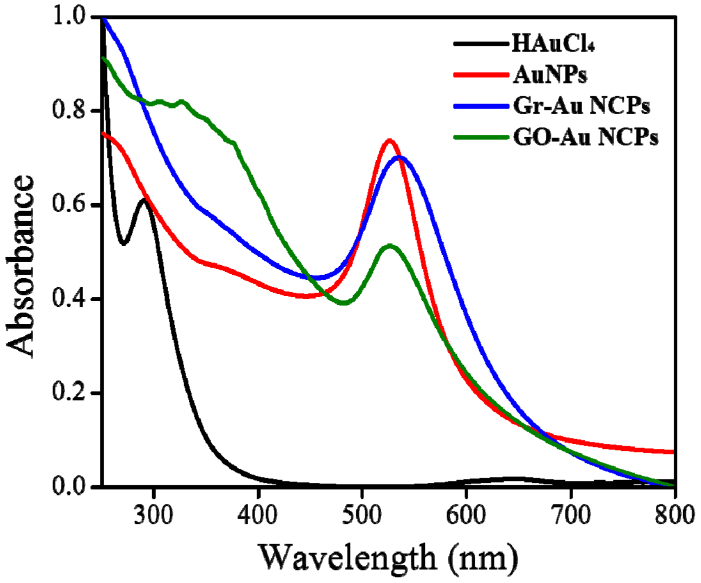

Figure S1. Characteristic absorption spectra of $HAuCl_4$ when used as an aqueous solution and the corresponding Au NPs, Gr/Au NCPs, and GO/Au NCPs.

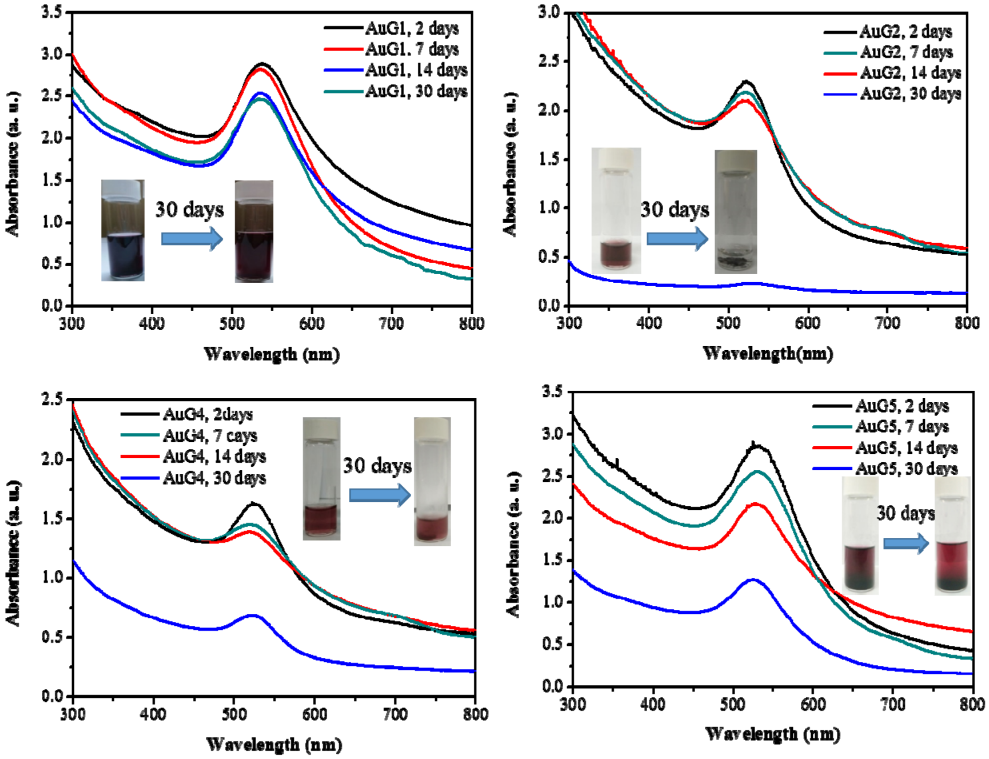

Figure S2. Characteristic absorption spectra of the corresponding Gr/Au NCPs by observing the absorption spectra of samples at different time intervals at room temperature. The inset shows the photo of the solution after 30 day storage at dark room.

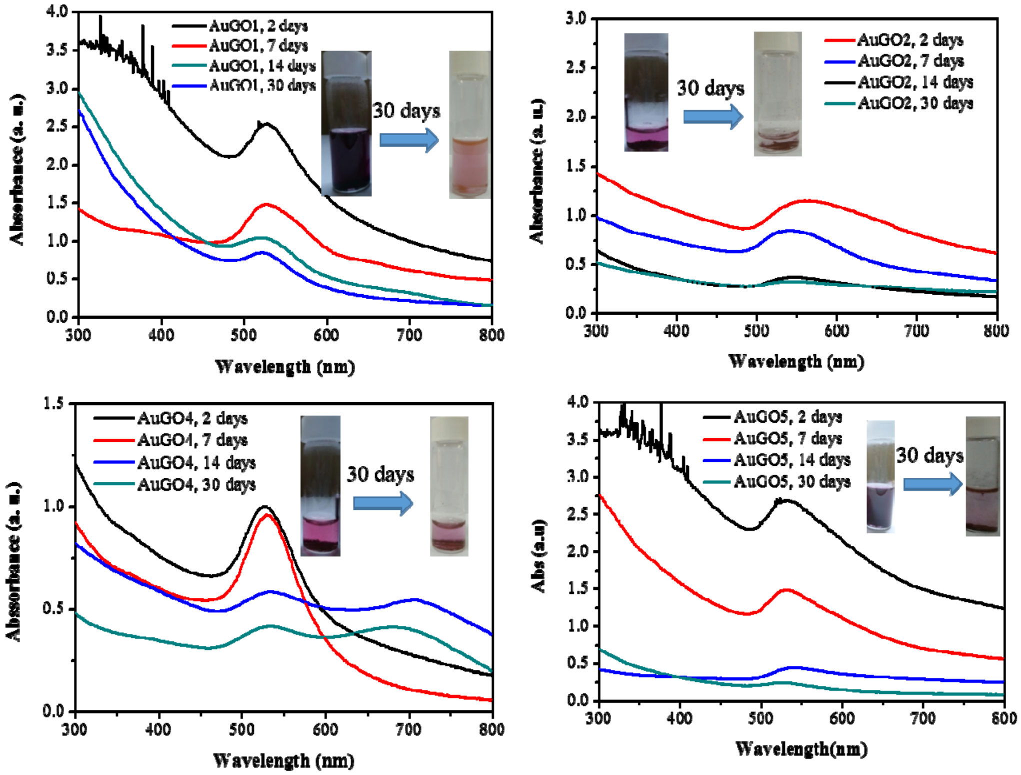

Figure S3. Characteristic absorption spectra of the corresponding GO/Au NCPs by observing the absorption spectra of samples at different time intervals at room temperature. The inset shows the photo of the solution after 30 day storage at dark room.

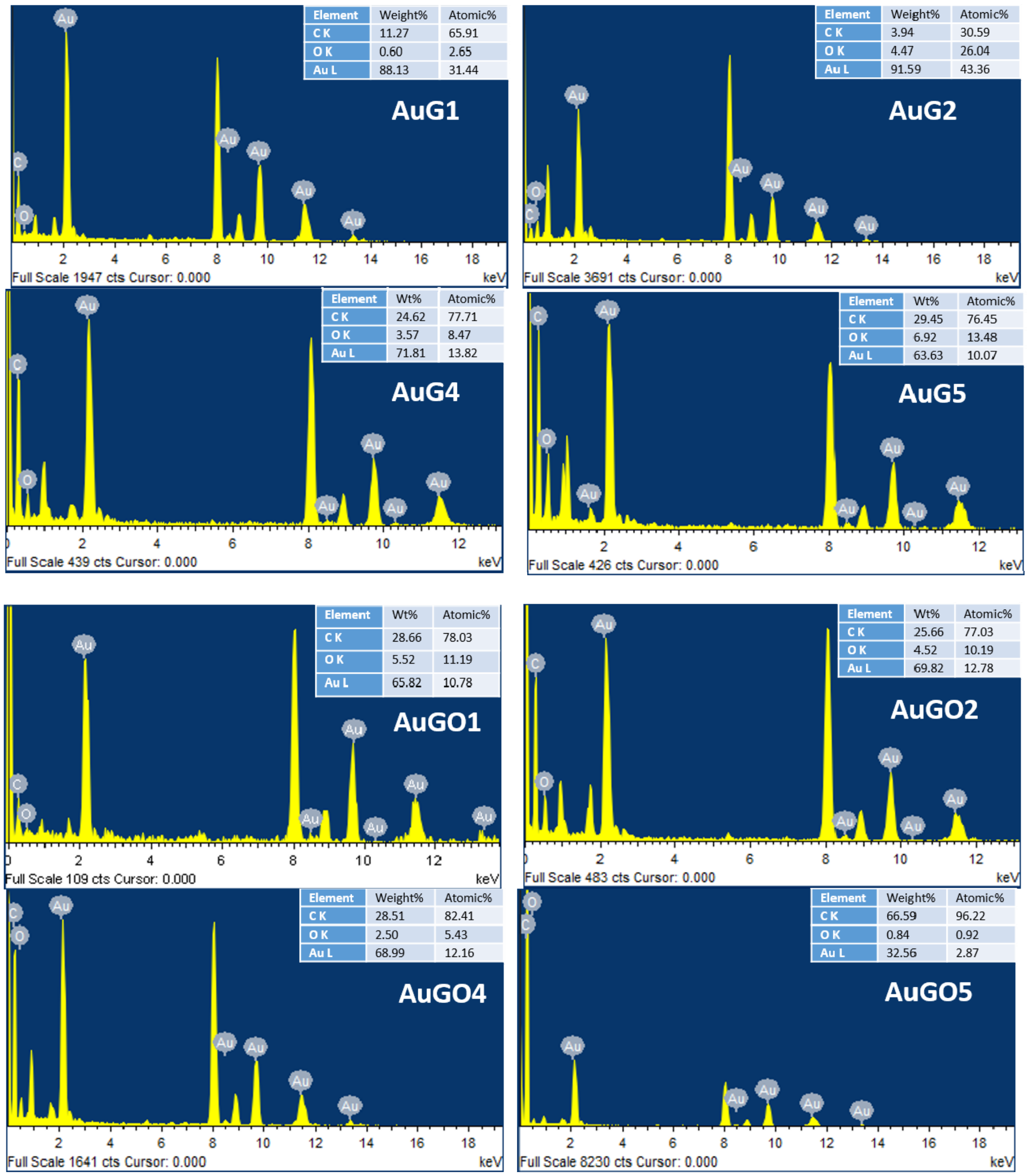

Figure S4. Elementary analysis of Gr/Au and GO/Au NCPs.

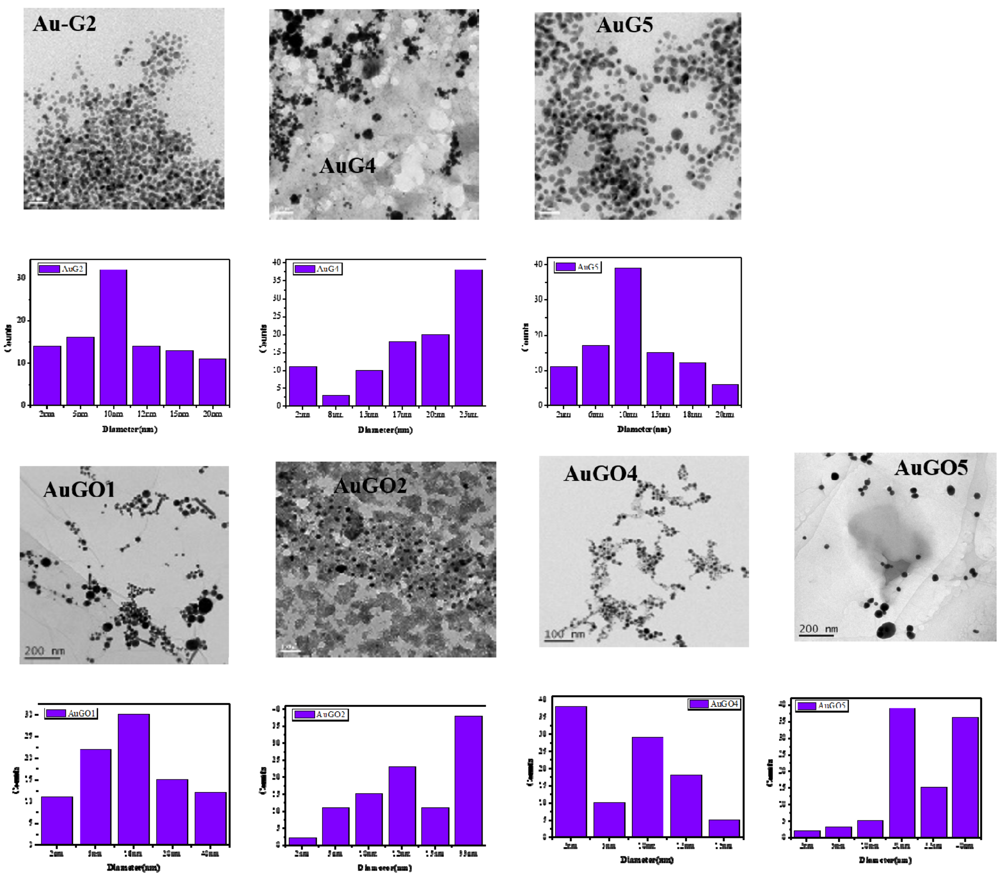

Figure S5. TEM image and corresponding size distribution of Gr/Au and GO/Au NCPs.

Table S2. Theoretical and experimental evaluation of Au loading.

Sample Number	Theoretical Gold Loading (vol%)	Real Gold loading (wt%)
AuG1	71	88.13
AuG2	50	91.59
AuG4	33	71.81
AuG5	83	63.63
AuGO1	71	65.82
AuGO2	50	69.82
AuGO4	33	68.99
AuGO5	83	32.56

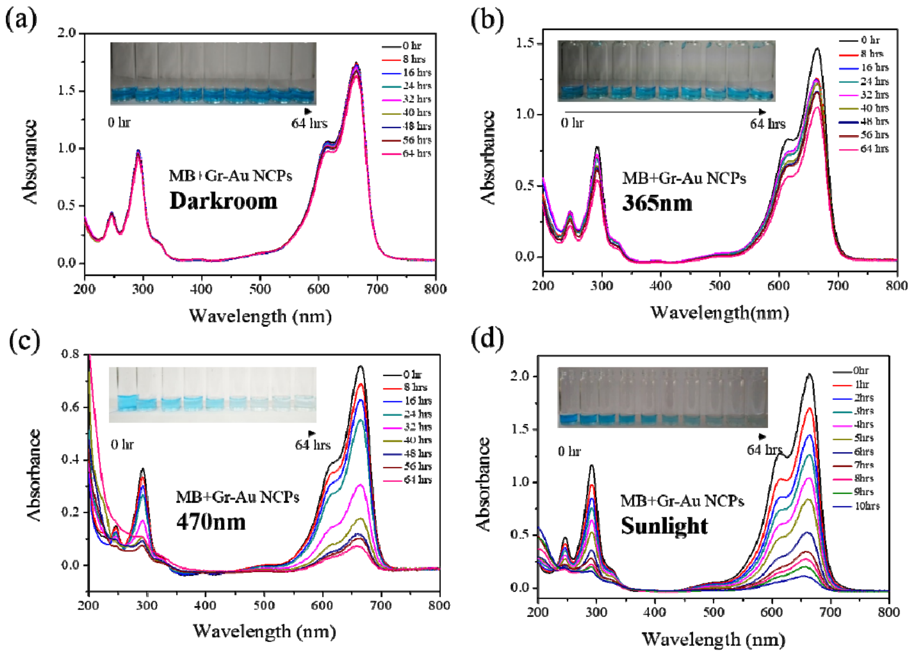

Figure S6. Change in absorbance of MB when photocatalytic degradation is performed (**a**) in a darkroom and under (**b**) UV, (**c**) visible, and (**d**) solar light in the presence of Gr/Au NCPs.

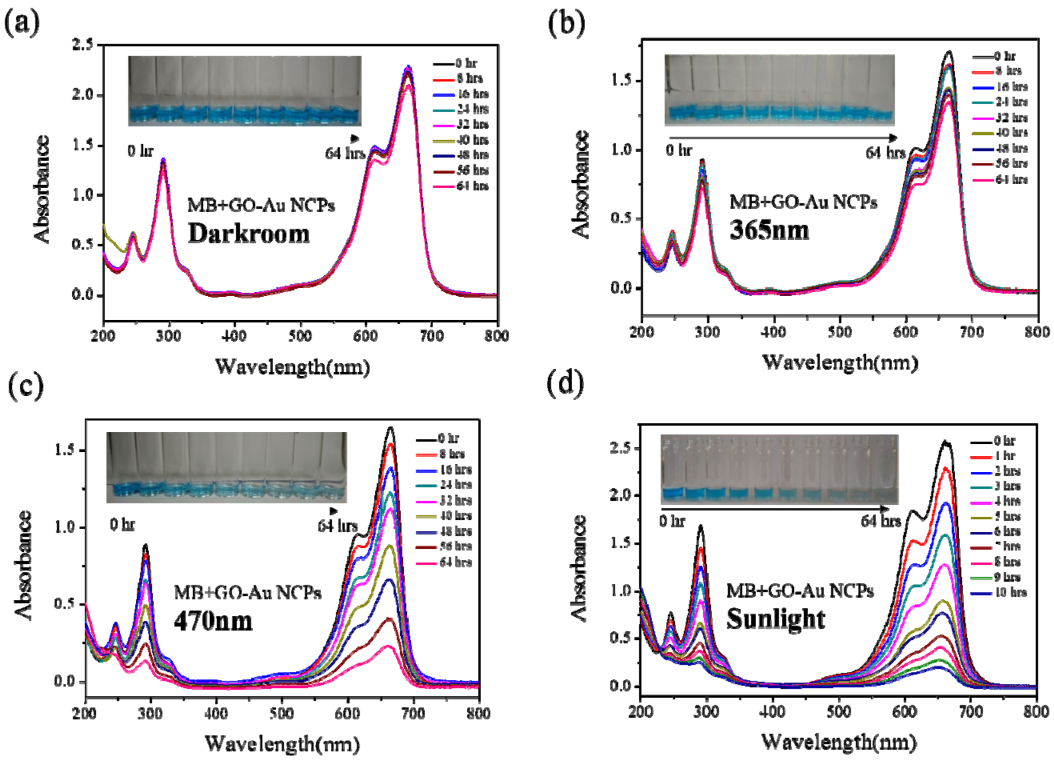

Figure S7. Change in the absorbance of MB when photocatalytic degradation is performed (**a**) in a darkroom and under (**b**) UV, (**c**) visible, and (**d**) solar lights in the presence of GO/Au NCPs.
